# Cutaneous Vulvar Tuberculosis Over an Episiotomy Scar in an Immunocompetent Patient

**DOI:** 10.7759/cureus.105516

**Published:** 2026-03-19

**Authors:** María Esther Suárez Garcia, Sorayda Mendez, Christopher Kaleb Romero Ríos, Lorenzo E Aragón Conrado, Francgiliz J Robleto, Catherine Scarlett Moreno Cabrera

**Affiliations:** 1 Obstetrics and Gynecology, Hospital Militar Escuela "Dr. Alejandro Dávila Bolaños", Managua, NIC; 2 School of Medicine, Hospital Militar Escuela "Dr. Alejandro Dávila Bolaños", Managua, NIC; 3 Medical Education, Escuela de Medicina Teniente Coronel y Doctor Sergio Martinez Ordoñez, Managua, NIC; 4 Medical Education, Hospital Militar Escuela "Dr. Alejandro Dávila Bolaños", Managua, NIC; 5 Internal Medicine, Hospital Militar Escuela "Dr. Alejandro Dávila Bolaños", Managua, NIC; 6 Research, Hospital Militar Escuela "Dr. Alejandro Dávila Bolaños", Managua, NIC

**Keywords:** cutaneous tuberculosis, episiotomy, granuloma, vulvar tuberculosis, ziehl–neelsen

## Abstract

Cutaneous tuberculosis is an uncommon extrapulmonary manifestation of *Mycobacterium tuberculosis* infection. Vulvar involvement is an exceptionally rare clinical entity that poses significant diagnostic challenges due to its similarity to other dermatologic and gynecologic conditions. We present the case of a 25-year-old immunocompetent woman with an 18-month history of a vulvar and perineal lesion that developed over the scar of a previous episiotomy. The patient was initially managed for a recurrent bacterial soft-tissue infection and received multiple antibiotic regimens without a therapeutic response. Upon admission, a raised, erythematous, granulomatous plaque with fistulization was observed. Definitive diagnosis was established through histopathological examination, which revealed chronic granulomatous inflammation and a Ziehl-Neelsen stain positive for acid-fast bacilli. To further complement the diagnostic workup, an interferon-gamma release assay was performed and was reactive; the tuberculin skin test was deferred due to the patient's prior BCG vaccination. Standard four-drug antituberculous therapy was initiated, resulting in a favorable clinical evolution. Vulvar tuberculosis should be considered in the differential diagnosis of chronic, ulcerated, or granulomatous genital lesions unresponsive to conventional antibiotic therapy, particularly in patients from endemic areas. Early clinical suspicion and timely biopsy are crucial to prevent morbidity associated with delayed diagnosis.

## Introduction

Despite being a preventable and curable disease, tuberculosis (TB) remains one of the leading causes of infectious morbidity worldwide. While pulmonary involvement is the classic presentation, extrapulmonary TB represents a significant diagnostic challenge. Within this spectrum, cutaneous tuberculosis (CTB) is an infrequent entity, comprising barely 1-2% of all extrapulmonary cases [1]. It encompasses several clinical forms, including tuberculosis verrucosa cutis (TVC), lupus vulgaris, and scrofuloderma. Vulvar involvement is an exceptional clinical rarity, representing less than 1% of all female genital TB cases [2].

Vulvar TB, in particular, poses a formidable challenge for gynecologists and dermatologists. Its clinical presentation is nonspecific and is often confused with sexually transmitted infections (STIs), deep mycoses, or malignancies, leading to prolonged diagnostic delays and ineffective empirical antibiotic treatments [3-5]. This report describes the case of a young, immunocompetent patient who developed cutaneous vulvar TB over a previous episiotomy scar, illustrating the phenomenon of locus minoris resistentiae and underscoring the importance of considering mycobacterial etiology in chronic, torpid cutaneous lesions in endemic areas.

## Case presentation

We present the case of a 25-year-old female patient with no significant chronic medical history, who presented for evaluation due to a long-standing clinical picture characterized by a lesion in the vulvar and perineal region. Significant obstetric history included a vaginal delivery 18 months prior to admission at a rural hospital, during which a right mediolateral episiotomy was performed. This surgical wound presented subsequent infectious complications that were managed on an outpatient basis with multiple antibiotic regimens without resolution, with symptoms persisting for a year and a half.

**Figure 1:**
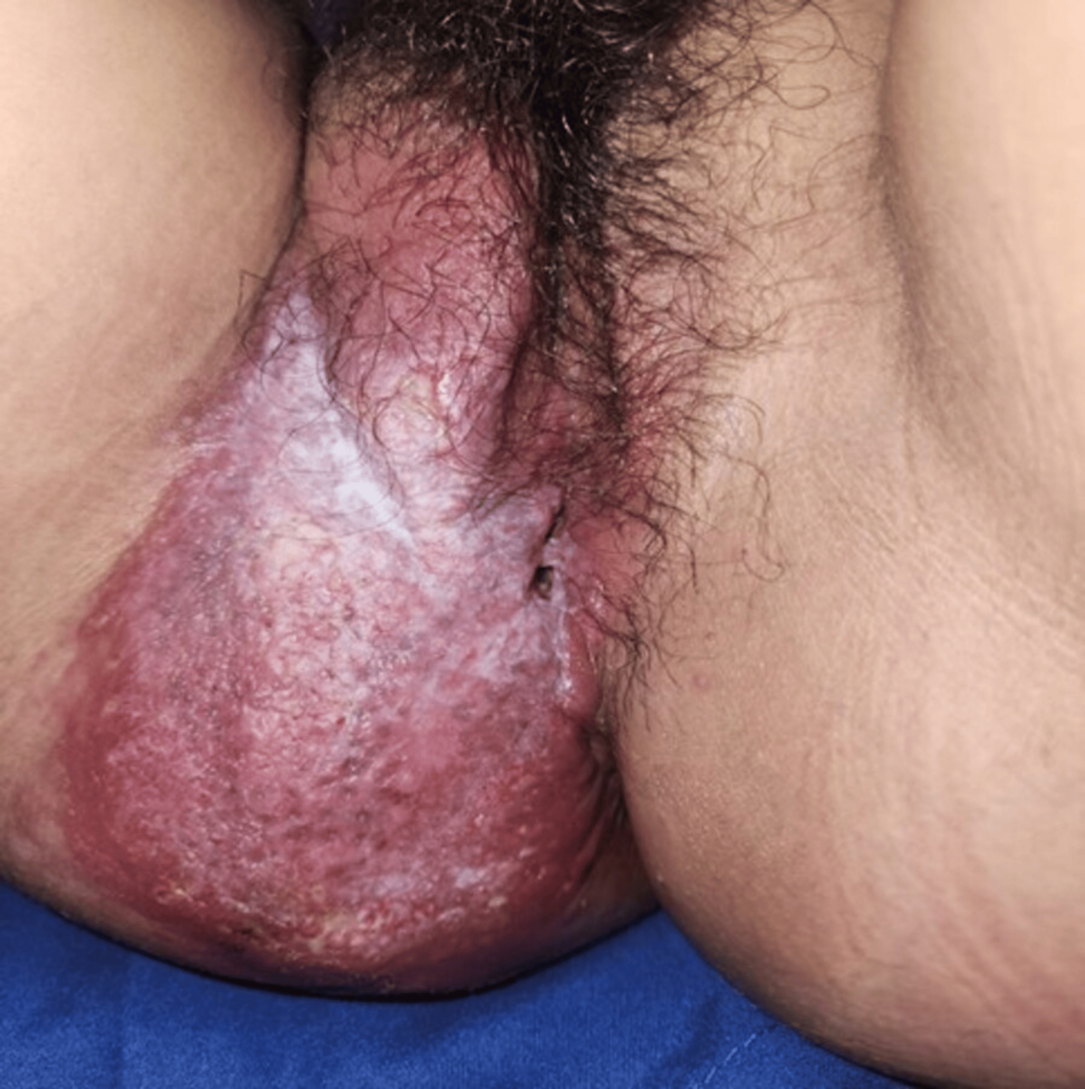
15 x 8 cm lesion encompassing the perineum.

Upon hospital admission, the patient had vital signs within normal parameters and denied systemic "B" symptoms such as fever, weight loss, night sweats, or persistent cough. Physical examination revealed a well-defined, raised, erythematous plaque (15 × 8 cm) with a fibroelastic consistency. The lesion spanned from the right labium majus to the intergluteal region, featuring a 4 mm fistula at the posterior fourchette with scant serous discharge. Notably, the lesion was painless upon palpation, and no inguinal lymphadenopathy was detected (Figure 1).

Initial laboratory studies revealed WHO grade II anemia, with hemoglobin at 8.3 g/dL, which subsequently rose to 11.7 g/dL following supplementation, as well as a significant elevation of C-reactive protein (249 mg/dL) and erythrocyte sedimentation rate (65 mm/hr) as an inflammatory marker (Table 1). To rule out common comorbidities and differential diagnoses, HIV screening and VDRL tests were performed, both of which were negative. Additionally, a chest X-ray showed no evidence of active or old pulmonary foci, suggesting a localized extrapulmonary presentation. Soft tissue ultrasound showed increased thickness and echogenicity of the subcutaneous tissue, with a cobblestone pattern compatible with edema and no evidence of drainable collections. An initial diagnosis of a complicated, chronic bacterial soft-tissue infection was suspected. Inpatient management was initiated with broad-spectrum antibiotics, including piperacillin/tazobactam, tigecycline, and cefepime, as well as antifungal coverage with voriconazole and daily local dressings with silver sulfadiazine and mupirocin.

**Table 1:** Laboratory values.

Laboratory Test	Patient Result	Reference Range	Units
Hemoglobin	8.3	12.0 – 16.0	g/dL
Hematocrit	25.1	36.0 – 46.0	%
White Blood Cells (Leukocytes)	15,400	4,500 – 11,000	cells/µL
Neutrophils	78	40 – 60	%
Lymphocytes	18	20 – 40	%
Platelets	4,10,000	150,000 – 450,000	cells/µL
C-Reactive Protein (CRP)	24.9	< 1.0	mg/dL
Erythrocyte Sedimentation Rate (ESR)	65	0 – 20	mm/hr

**Figure 2:**
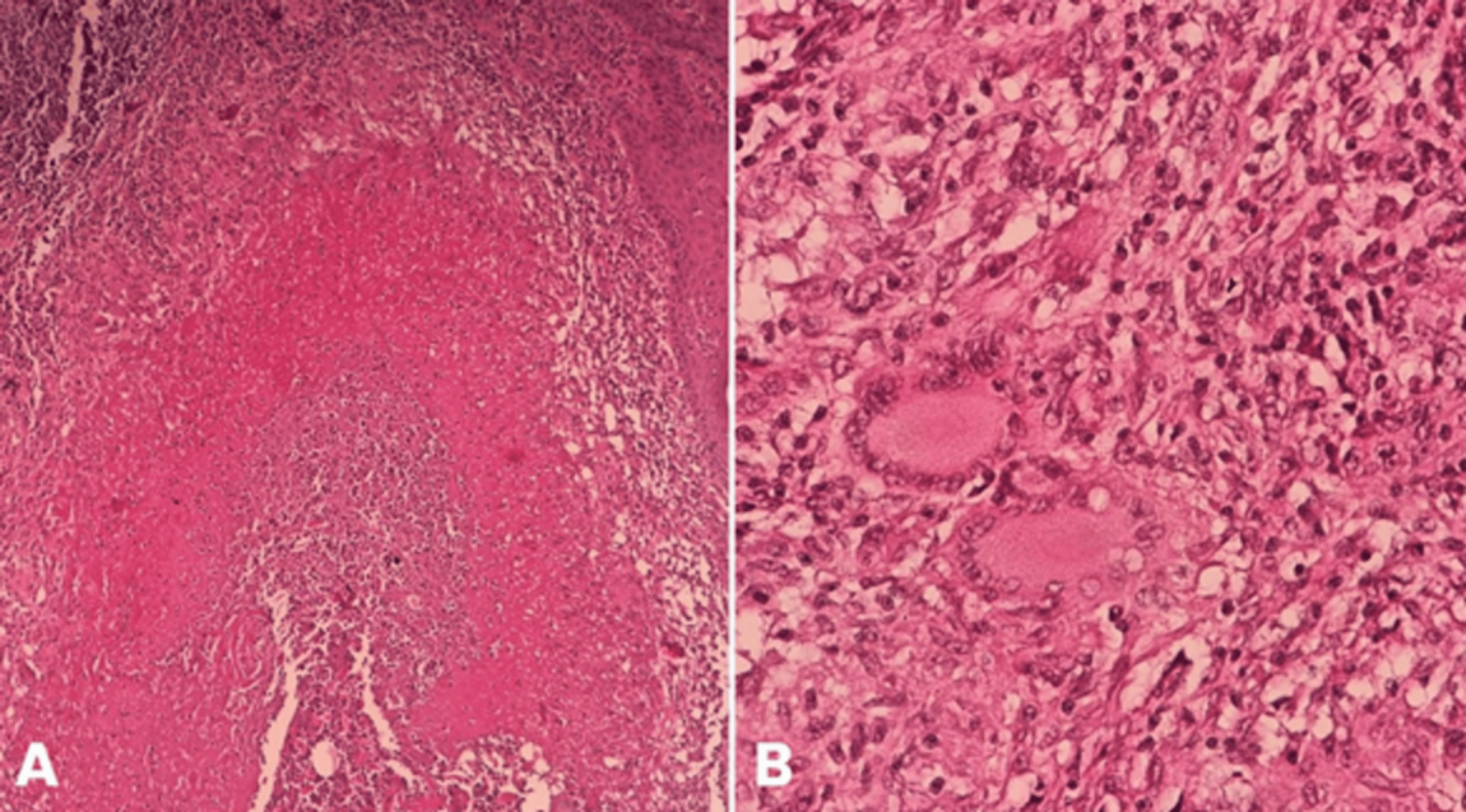
Histopathology of the tissue. A (H&E, 10x): Multiple granulomas with central caseous necrosis, surrounded by lymphocytes and epithelioid macrophages, with stromal edema and loss of normal architecture. B (H&E, 40x): Granulomas with epithelioid macrophages and Langhans-type giant cells; central caseous necrosis and mixed lymphoplasmacytic infiltrate.

Due to this lack of therapeutic response, a definitive diagnostic workup was pursued through a tissue biopsy. The histopathological report described chronic caseating granulomatous inflammation; special stains (PAS and Grocott) were negative for fungal elements, ruling out deep mycoses and malignancy. Notably, the Ziehl-Neelsen stain was positive for acid-fast bacilli (AFB ++) (Figure 2). To "close the circle" and provide immunological confirmation of the infection, an interferon-gamma release assay (IGRA - QuantiFERON-TB Gold) was performed and was reactive. A tuberculin skin test (PPD) was deferred because the patient’s history of BCG vaccination would have compromised the test’s specificity.

**Figure 3:**
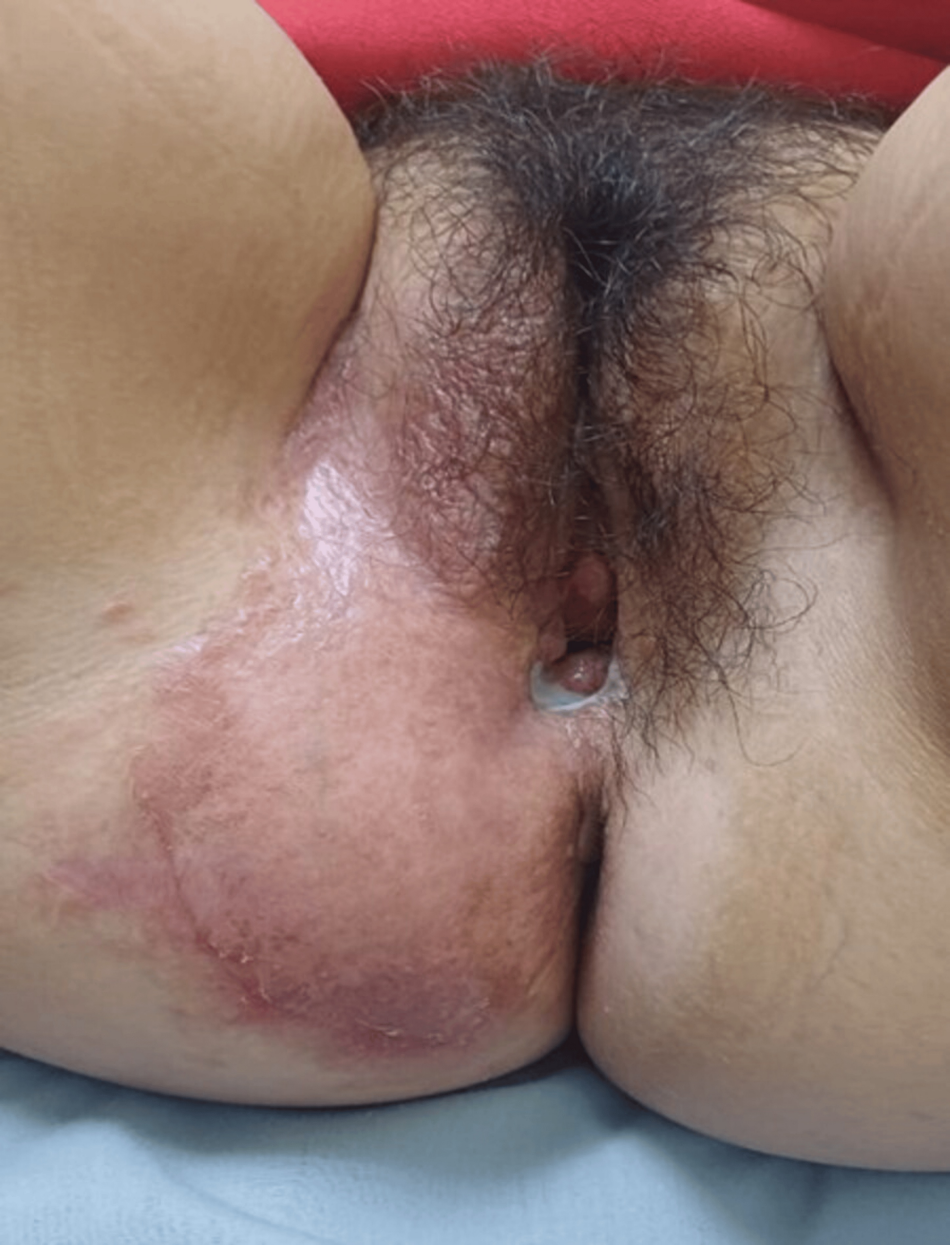
Clinical improvement of the lesion at three months.

Following the diagnosis, a contact investigation was initiated. The patient’s sexual partner and her 18-month-old child (born at the time of the episiotomy) were referred for clinical evaluation and PPD testing; both remained asymptomatic. The patient began the standard four-drug regimen (RIPE). At the three-month follow-up, the lesion showed significant flattening, and the fistula had completely closed (Figure 3). The patient is scheduled to complete the full six-month course with a final microbiological assessment to confirm objective cure.

## Discussion

CTB is an uncommon clinical manifestation caused by *Mycobacterium tuberculosis*. It is so rare that vulvar localization is considered an exceptional clinical entity, comprising barely 1-2% of all extrapulmonary cases [1]. Vulvar involvement is an exceptional clinical rarity, representing less than 1% of all female genital TB cases [2]. Recent reports continue to highlight the diagnostic challenges of female genital TB, which often presents with non-specific symptoms or mimics masses, leading to significant delays in management [3,4]. The presented case stands out due to its appearance over a previous surgical scar, mimicking a resistant surgical site bacterial infection. The initial administration of broad-spectrum antibiotics was a response to the high inflammatory burden (CRP 24.9 mg/dL) and the lesion's appearance; however, the lack of therapeutic response served as a diagnostic clue to pivot toward mycobacterial etiologies.

The diagnosis of vulvar TB is notoriously difficult due to its behavior as "the great imitator." In our patient, the 18-month interval between symptom onset and definitive diagnosis-a period during which the patient sought care at multiple facilities without reaching a correct diagnosis-is consistent with the diagnostic delays reported in the literature [5,6]. Lesions are often initially misdiagnosed as STIs, mycetomas, or squamous cell carcinomas [7,8]. In this case, a systematic exclusion of differential diagnoses was performed: STIs were ruled out by negative serology; malignancy was excluded by the absence of cellular atypia or basement membrane invasion in the histopathology; and mycetoma was discarded due to negative PAS and Grocott stains along with the failure of empirical antifungal coverage.

Because CTB is typically paucibacillary, conventional microbiological methods such as AFB smears and cultures often yield low sensitivity in skin samples [9]. Therefore, a mandatory biopsy remains the gold standard for definitive diagnosis, as it allows for the histological identification of caseating granulomas and the detection of bacilli through special stains [9,10]. In our patient, the histopathological findings were the primary diagnostic driver. To complement this workup and provide immunological confirmation, an IGRA was performed and was reactive; the tuberculin skin test (PPD) was deferred due to the patient's history of BCG vaccination, which limits the test's specificity.

From a pathophysiological perspective, the history of a previous episiotomy suggests a mechanism of direct inoculation or seeding in vulnerable tissue, known as locus minoris resistentiae [11]. This phenomenon is well-documented in areas of local trauma [11,12]. Although the ulcerative form is widely recognized as the most common presentation of vulvar TB [13,14], our patient presented a hypertrophic variant with verrucous characteristics, which is usually associated with TVC in patients with preserved cellular immunity [7].

Standard antituberculous therapy demonstrated high efficacy, leading to complete symptom remission and lesion healing. This outcome aligns with previously reported cases of successful therapeutic response following a standard regimen [8]. However, the 18-month delay in reaching our specialized unit underscores the significant morbidity associated with clinical oversight in chronic vulvar lesions. Early clinical suspicion and timely biopsy are essential to prevent extensive tissue destruction and long-term morbidity.

## Conclusions

The presentation of CTB in the vulvar region constitutes a significant diagnostic challenge due to its low incidence and ability to mimic other inflammatory, infectious, or neoplastic conditions. This case illustrates how a history of local surgical trauma, such as an episiotomy, can act as a predisposing factor for the development of infection through the locus minoris resistentiae phenomenon, complicating the initial clinical picture and diverting diagnostic suspicion toward common bacterial etiologies.

It is imperative that medical personnel maintain a high index of suspicion for long-standing genital cutaneous lesions that present resistance to conventional treatment. Clinical reliance alone is insufficient; therefore, tissue biopsy for histopathological studies and specific staining is established as the gold standard for definitive diagnosis. Early recognition of this pathology allows for the timely initiation of specific antituberculous treatment, which, as demonstrated in this report, leads to an excellent prognosis and complete resolution of lesions, avoiding major complications and long-term anatomical sequelae.
